# Systemic immune inflammation index in patients with recurrent aphthous stomatitis

**DOI:** 10.1016/j.bjorl.2022.02.007

**Published:** 2022-03-21

**Authors:** Fatma Atalay, Ayhan Kars, Kubra Topal, Zeynep Yavuz

**Affiliations:** a Kastamonu University, Faculty of Medicine, Department of Otorhinolaryngology, Kastamonu, Turkey; b Ankara University, Faculty of Medicine, Department of Biostatistics, Ankara, Turkey

**Keywords:** Inflammatory marker, Hematinic deficiencies, Systemic immune inflammation index, Systemic inflammation, Recurrent aphthous stomatitis

## Abstract

**Objectives:**

Recurrent Aphthous Stomatitis (RAS) a chronic idiopathic oral mucosal disease. But yet the etiology and pathogenesis of RAS are not exactly known, it is thought that inflammation play an important role in the pathogenesis. The aim of this study is to demonstrate the role of systemic inflammation among the possible etiological factors of RAS and to find the possible diagnostic correlation between Systemic Immune Inflammation Index (SII).

**Methods:**

Patients who were consulted the otolaryngology outpatient clinic and diagnosed with RAS between 2019–2021 were retrospectively analyzed. Neutrophil/Lymphocyte Ratio (NLR), Platelet/Lymphocyte Ratio (PLR) and SII values were calculated based on the results of complete blood count. Demographic and hematological parameters between control and RAS groups were compared. The statistical significance level was considered as <0.05.

**Results:**

There was no statistically significant difference between the control and RAS groups in terms of sex and age distributions (*p* = 0.566 and *p* = 0.173, respectively). SII, NLR and PLR values were significantly higher in the RAS group compared to the controls (*p* < 0.001, *p* < 0.001 and *p* = 0.001, respectively). A very strong correlation between SII and NLR, moderately strong correlation between SII and PLR and moderate correlation between NLR and PLR values were detected (respectively *ρ*: 0.813, 0.719, 0.532; *p*-values <0.001).

**Conclusion:**

SII, NLR and PLR has significantly higher levels in the RAS group compared to the control group, that it supports the role of systemic inflammation in the etiopathogenesis of RAS. In addition, the results show that SII is a valuable marker for inflammation.

**Level of evidence:**

4.

## Highlights

• RAS is a chronic, idiopathic, ulcerative oral mucosal disease.
• SII is a new and inexpensive biomarker that can easily be calculated using the platelet, neutrophil, and lymphocyte count.
• SII may be a valuable marker to demonstrate the role of systemic inflammation in RAS etiopathogenesis.
• Vascular, thrombotic, and inflammatory processes are thought to have a role in RAS activation.

## Introduction

Recurrent Aphthous Stomatitis (RAS) a chronic idiopathic oral mucosal disease which is an endemic, and characterized by painful recurrent ulcers.^1^ They are most commonly located in the buccal mucosa, the lower surface of the tongue and the floor of the mouth.^2^ The most common form (approximately 80%) of RAS, which has 3 clinical forms as major, minor and herpetiform, is the minor form.^3^ Painful ulcers affect negatively the quality of life of the person because of causing difficulty in eating and speaking. In general, they heal spontaneously within 2 weeks without scarring.^1^, ^2^

But yet the etiology and pathogenesis of RAS are not exactly known, it is considered as a multifactorial process caused by various triggering factors and immunological disorders. Possible triggers may be listed hematinic deficiencies such as ferritin, vitamin B12, folate deficiency, stress and anxiety, mucosal trauma, food allergies, genetic predisposition, connective tissue diseases and hormonal changes.^1^, ^2^, ^3^, ^4^ Besides, oxidative stress caused by systemic inflammation is thought to play a role in the etiopathogenesis of RAS.^1^ By reason of the fact that the pathogenesis is unclear, its treatment is symptomatic.

Blood-based inflammation markers have a great role in demonstrating systemic inflammation. Neutrophil/Lymphocyte Ratio (NLR), Platelet/Lymphocyte Ratio (PLR) and Mean Platelet Volume (MPV) are recently widely used inflammation markers. The Systemic Immune Inflammation Index (SII), which was calculated by using the formula of “neutrophil count × platelet count/lymphocyte count” by Hu et al. in 2014, is a new inflammation maker, which has been ever increasing use in recent years.^5^, ^6^

The aim of this study is to demonstrate the role of systemic inflammation among the possible etiological factors of RAS and to find the possible diagnostic correlation between SII, a new inflammatory marker, and NLR and PLR.

## Methods

Ethical approval was received from Kastamonu University Clinical Research Ethics Committee in order to conduct the study (Decision nº 2020-KAEK-143-30, Date: 28.01.2021). All procedures performed in studies involving human participants were in accordance with the ethical standards of the institutional (Kastamonu University Clinical Research Ethics Committee) and with the 1964 Helsinki declaration and its later amendments or comparable ethical standards.

In our study, patients who were consulted the otolaryngology outpatient clinic and diagnosed with RAS between 2019–2021 were retrospectively analyzed. An age and gender matched healthy control group was formed with the study group. Those with heart diseases such as congestive heart failure, valvular heart disease or myocardial infarction, those with autoimmune diseases such as Hashimoto’s thyroiditis, Behçet’s disease, those with findings of infection such as white blood cell count >12,000 mL, neu >70%, those with hematological disease hemoglobin level <12 g/dL or >18 g/dL, those with sickle cell anemia, patients with coagulopathy such as factor 5 Leiden mutation were not included in the study. As a result, 97 patients (52 females, 45 males), 97 healthy controls (48 females, 49 males) were included in the study.

NLR, PLR and SII values were calculated based on the results of complete blood count. NLR = Neutrophil count/Lymphocyte count, PLR = Platelet count/Lymphocyte count and SII = neutrophil count × platelet count/lymphocyte count formulas were used in the calculation.^7^

Descriptive statistics were presented as frequency (percentage) for categorical variables and mean ± standard deviation or median (minimum‒maximum) for quantitative variables depending on their compatibility with the normal distribution according to Kolmogov–Smirnov test and graphical methods. Demographic and hematological parameter comparisons between control and RAS groups were made using Mann–Whitney *U* Test for quantitative and Chi-Square for qualitative variables. Standardized differences (effect size ‒ Cohen’s *d*) between the two groups in terms of SII, NLR and PLR are presented. Relationships between the quantitative variables were evaluated with the Spearman correlation coefficient (ρ). Area Under the Receiver Operating Characteristic Curve (ROC) was used to assess the discrimination ability of SII, NLR and PLR values for RAS, and cut-off values were obtained using Youden’s Index and sensitivity – specificity values with their 95% Confidence Intervals were calculated. All statistical analyzes were performed with Statistical Package for Social Sciences, Version 15.0 (SPSS Inc. Chicago, IL, USA) and the statistical significance level was considered as <0.05.

## Results

There was no statistically significant difference between age and sex distributions of the RAS and control groups (*p* = 0.173, 0.566, respectively) (Table 1).

**Table 1:** Age and gender distribution by groups.

Variable^a^	Control (n = 97)	RAS (n = 97)	*p*-Value
Sex, n (%)			
Female	48 (49.5)	52 (53.6)	0.566
Male	49 (50.5)	45 (46.4)	
Age (years)	32 (18–60)	34 (18–55)	0.173

a Values are presented as Median (Minimum‒Maximum) unless stated otherwise.

SII, NLR and PLR values were significantly higher in the RAS group compared to the control (*p* < 0.001; *p* < 0.001 and *p* = 0.001, respectively) (Table 2).^8^, ^9^

**Table 2:** SII, NLR and PLR values of participants.

Variable^a^	Control (n = 97)	RAS (n = 97)	Effect size (Cohen’s d)	*p-*Value
SII	409.8 (121.4–831.7)	506.0 (144.8–1448.3)	0.589	<0.001
NLR	1.54 (0.63–3.10)	1.86 (0.72–6.22)	0.550	<0.001
PLR	105.9 (18.2–284.8)	125.3 (64.9–303.7)	0.506	0.001

Effect size cut-off values; 0.2: Small, 0.5: Medium, 0.8: Large, 1.3: Very Large. Cohen (1988) & Rosenthal (1996).^8^, ^9^

a Values are presented as Median (Minimum‒Maximum).

A very strong correlation between SII and NLR within the RAS group was observed followed with a moderately strong correlation between SII and PLR and a moderate correlation between NLR and PLR values (respectively, *ρ*: 0.813; 0.719; 0.532, *p-*values < 0.001) (Table 3).^10^

**Table 3:** Correlations between SII, NLR and PLR values in the RAS group.

Variables	*ρ*	*p*-Value
SII ‒ NLR	0.813	<0.001
SII ‒ PLR	0.719	<0.001
NLR ‒ PLR	0.532	<0.001

Values ≥0.8 indicate a very strong, 0.6 up to 0.8 ‒ moderately strong, 0.3 to 0.5 – fair, less than 0.3 – poor to no correlation.^10^

**Figure 1:**
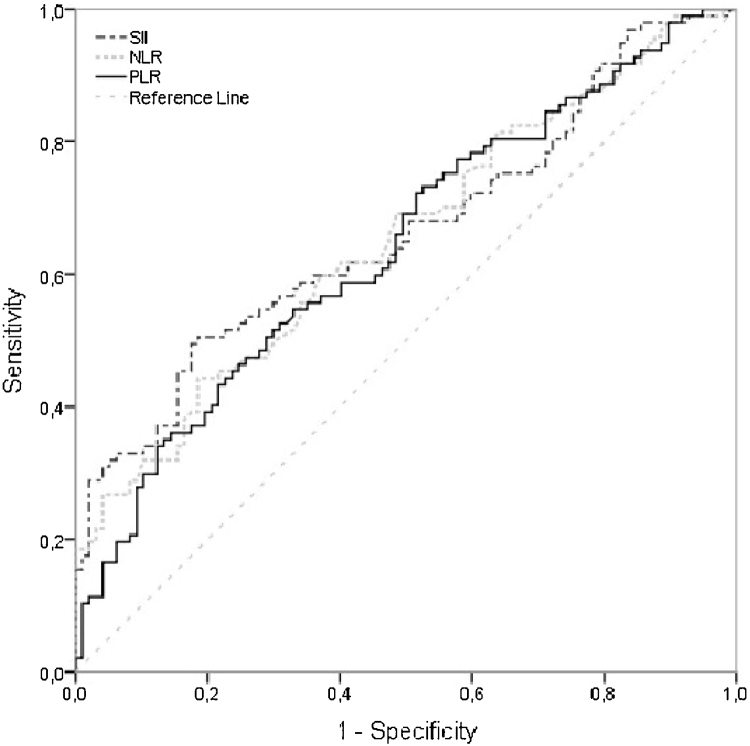
ROC curves for SII, NLR and PLR.

SII, NLR and PLR values were found to be significantly higher in the RAS group however the AUC values regarding these indexes, while being slightly sigher for SII, were relatively low [AUC (95% CI) respectively; 0.663 (0.587‒0.740), 0.653 (0.577‒0.730), 0.642 (0.565‒0.719)] to derive a value that above values indicate inflammation related RAS. Hence cut-off values for SII, NLR and PLR resulted in a low sensitivity while being highly specific (Table 4, Fig. 1).^11^

**Table 4:** Area under the ROC curve, sensitivity, and specificity values for SII, NLR and PLR.

Variable	AUC (95% CI)^a^	*p*-Value	Cut-off value	Sens (95% CI)	Spec (95% CI)
SII	0.663 (0.587‒0.740)	<0.001	≥514	0.495 (0.392‒0.598)	0.825 (0.734‒0.895)
NLR	0.653 (0.577‒0.730)	<0.001	≥2	0.443 (0.342‒0.548)	0.814 (0.723‒0.886)
PLR	0.642 (0.565‒0.719)	0.001	≥140	0.351 (0.256‒0.454)	0.866 (0.781‒0.923)

AUC, Area Under the Curve, CI: Confidence Interval, Sens: Sensitivity, Spec: Specificity.

a AUC = 0.5 no discrimination, 0.7 ≤ AUC < 0.8 ‒ acceptable discrimination, 0.8 ≤ AUC < 0.9 ‒ excellent discrimination, AUC ≥ 0.9 outstanding discrimination.^11^

## Discussion

RAS is an epidemic ulcerative oral mucosal disease affecting approximately 10%‒20% of the population. Although it is known that potential triggers play a role for most factors such as nutrition, hormonal changes, allergy, genetic, psychological, traumatic, and infectious, the etiopathogenesis of RAS has not been exactly clarified yet. Nonetheless, it is thought that immunological mechanisms and oxidative stress mediated inflammation play an important role in the pathogenesis.^1^, ^12^

There are studies showing that vascular, thrombotic, and inflammatory processes have a role in RAS activation. Endothelial dysfunction, chronic inflammation and thrombosis trigger the formation of aphthous lesions.^2^ Neutrophils and lymphocytes are responsible for the subclinical inflammation process, and platelet dysfunction is responsible for thrombosis. NLR and PLR are recently frequently used markers indicating the presence and severity of subclinical inflammation. The increased level of NLR and PLR in RAS patients indicates that vascular, thrombotic, and inflammatory processes play a role in RAS activation. It is also known that the increase in NLR correlates with the increase in disease activity.^2^, ^12^ In our study, NLR and PLR were found to be statistically significantly higher in RAS patients as supporting the literature.

It is also thought that aphthous lesions develop in response to an increased immunological reaction against certain areas of the oral mucosa in individuals with a genetic predisposition. There are studies supporting the important role of immunological disorders in the etiopathogenesis of RAS.^13^ It has been found that the immune system function is disabled in response to triggering factors in patients with RAS and the secretion of anti-inflammatory cytokines is significantly reduced in RAS patients compared to healthy individuals. It is thought that this imbalance in the production of pro and anti-inflammatory cytokines has caused autoimmunization and RAS development in predisposition individuals.^14^

SII is a new and inexpensive biomarker that can easily be calculated using the platelet, neutrophil, and lymphocyte count, showed the balance between inflammatory and immune responses. SII has been researched as a systemic inflammation and prognostic marker in many malignant diseases, vasculitis, Bell’s palsy, nasal polyps, and asthma. High SII values have been correlated with poor prognosis in malignant diseases and generally indicate a strong inflammatory response and a weak immune response.^5^, ^15^, ^16^ In the studies of asthma and Bell’s palsy, it was concluded that SII reflects the inflammatory etiology of the disease better than other systemic inflammation markers.^5^, ^15^

In our study, SII, NLR and PLR values were found to be statistically significantly higher in the RAS group compared to the healthy control group. In addition, it was found that SII was highly correlated with NLR and PLR values within the RAS group. The results support the role of systemic inflammation in the etiopathogenesis of RAS and show that SII is a valuable marker for inflammation.

As far as we know, our study is the first study in the literature showing the role of SII in RAS patients. Due to being retrospective, the limitations of our study are that we do not have data regarding the severity of the disease and the frequency of recurrence of aphthae in RAS patients and that we calculated SII for only one application of the patients. For this reason, no results could have been obtained related to the prognostic role of SII and its change in the process. More studies are needed on this topic.

## Conclusion

SII, NLR and PLR has a significantly higher levels in the RAS group compared to the control group, that it supports the role of systemic inflammation in the etiopathogenesis of RAS.

## Data availability

The data that has been used is confidential.

## Conflicts of interest

The authors declare no conflicts of interest.
